# Association between pain interference and motoric cognitive risk syndrome in older adults: a population-based cohort study

**DOI:** 10.1186/s12877-024-04974-7

**Published:** 2024-05-17

**Authors:** Gege Li, Zijun He, Jinjing Hu, Chongwu Xiao, Weichao Fan, Zhuodong Zhang, Qiuru Yao, Jihua Zou, Guozhi Huang, Qing Zeng

**Affiliations:** 1 Department of Rehabilitation Medicine, Zhujiang Hospital, Southern Medical University, Guangzhou, China; 2 School of Rehabilitation Medicine, Southern Medical University, Guangzhou, China; 3 Department of Rehabilitation Medicine, The Tenth Affiliated Hospital of Southern Medical University (Dongguan people’s hospital), Dongguan, China; 4 School of Nursing, Southern Medical University, Guangzhou, China; 5 Department of Rehabilitation Sciences, The Hong Kong Polytechnic University, Hong Kong (SAR), China

**Keywords:** Pain interference, Older adults, Motoric cognitive risk syndrome, Cohort

## Abstract

**Objectives:**

Motoric cognitive risk syndrome (MCR) is a pre-dementia condition characterized by subjective complaints in cognition and slow gait. Pain interference has previously been linked with cognitive deterioration; however, its specific relationship with MCR remains unclear. We aimed to examine how pain interference is associated with concurrent and incident MCR.

**Methods:**

This study included older adults aged ≥ 65 years without dementia from the Health and Retirement Study. We combined participants with MCR information in 2006 and 2008 as baseline, and the participants were followed up 4 and 8 years later. The states of pain interference were divided into 3 categories: interfering pain, non-interfering pain, and no pain. Logistic regression analysis was done at baseline to examine the associations between pain interference and concurrent MCR. During the 8-year follow-up, Cox regression analysis was done to investigate the associations between pain interference and incident MCR.

**Results:**

The study included 7120 older adults (74.6 ± 6.7 years; 56.8% females) at baseline. The baseline prevalence of MCR was 5.7%. Individuals with interfering pain had a significantly increased risk of MCR (OR = 1.51, 95% CI = 1.17–1.95; *p* = 0.001). The longitudinal analysis included 4605 participants, and there were 284 (6.2%) MCR cases on follow-up. Participants with interfering pain at baseline had a higher risk for MCR at 8 years of follow-up (HR = 2.02, 95% CI = 1.52–2.69; *p* < 0.001).

**Conclusions:**

Older adults with interfering pain had a higher risk for MCR versus those with non-interfering pain or without pain. Timely and adequate management of interfering pain may contribute to the prevention and treatment of MCR and its associated adverse outcomes.

**Supplementary Information:**

The online version contains supplementary material available at 10.1186/s12877-024-04974-7.

## Introduction

Dementia is a neurodegenerative disease [1]. Before the onset of mild cognitive impairment (MCI), there may be a long preclinical stage that persists for several years to decades [2]. Both subjective memory complaints and slow gait are potent independent indicators of cognitive deterioration and dementia [3, 4]. Throughout this extended preclinical stage, slower gait and subjective memory complaints typically manifest concurrently [5]. The motoric cognitive risk syndrome (MCR), which integrates these two early indicators of dementia [6–8], has been reported to be a stronger predictor of cognitive decline compared to either of them alone [9]. It is considered an intermediary stage transitioning from normal aging to MCI. The prevalence of MCR is 9.7% among older adults according to a multi-country analysis from 22 cohorts [7]. In addition to dementia, MCR has been reported as a risk factor for various negative outcomes such as physical impairment [10], mortality [11], and falls [12]. Thus, to promote healthy and active aging, it is necessary to identify the modifiable risk factors for MCR.

Pain affects more than 20% of the community-living older adults [13]. Pain is known to be related to geriatric syndromes including cognitive decline and dementia [14], falls [15], and functional disability [16]. Chronic diseases caused by pain such as sarcopenia [13], cardiovascular diseases [17], and depression [18] may become risk factors for MCR [5]. Longitudinal studies have shown that the presence and intensity of pain were predictors of incident MCR [19, 20]. Individuals who experience severe pain are five times more likely to develop MCR than those without pain [20]. However, pain is a multidimensional and subjective experience [21, 22]. Studies examining the associations between MCR and other pain characteristics, such as pain interference, are limited.

Pain interference provides information about the impact of pain on daily activities. Compared to pain intensity, it holds equal or even superior significance. Even among individuals with similar pain intensity levels, their pain interference considerably varies [23]. Recent studies suggest that pain interference may be a stronger predictor of cognitive decline compared to pain intensity [22, 24, 25]. Interfering pain is associated with the key characteristics that define MCR. A previous study found an association between interfering pain and poorer memory as well as executive function [26], which is relied upon for walking [27]. In addition, interfering pain may impair complex attention, which is vital for the daily function and mobility of older adults [28]. Thus, interfering pain may be closely related to MCR.

To investigate the cross-sectional and longitudinal associations between different states of pain interference and MCR, baseline data and 8 years of follow-up data were obtained from an older population aged ≥ 65 years in the Health and Retirement Study (HRS). The states of pain interference were divided into three categories: interfering pain, non-interfering pain, and no pain. Moreover, we conducted three types of sensitivity analyses to investigate the robustness of the association. We hypothesized that the risk of MCR is increased in individuals with interfering pain compared with those with non-interfering pain or without pain. This study may contribute to the prevention and the establishment of intervention outcomes for pre-dementia syndrome. Additionally, the assessment of MCR does not require trained personnel or specialized equipment [20], which facilitates more frequent and regular cognitive function monitoring during both clinical-based and home-based pain management. Ultimately, it helps to evaluate the effectiveness of pain management measures, and to guide and assess the treatment plan.

## Methods

### Study design and population

**Fig. 1:**
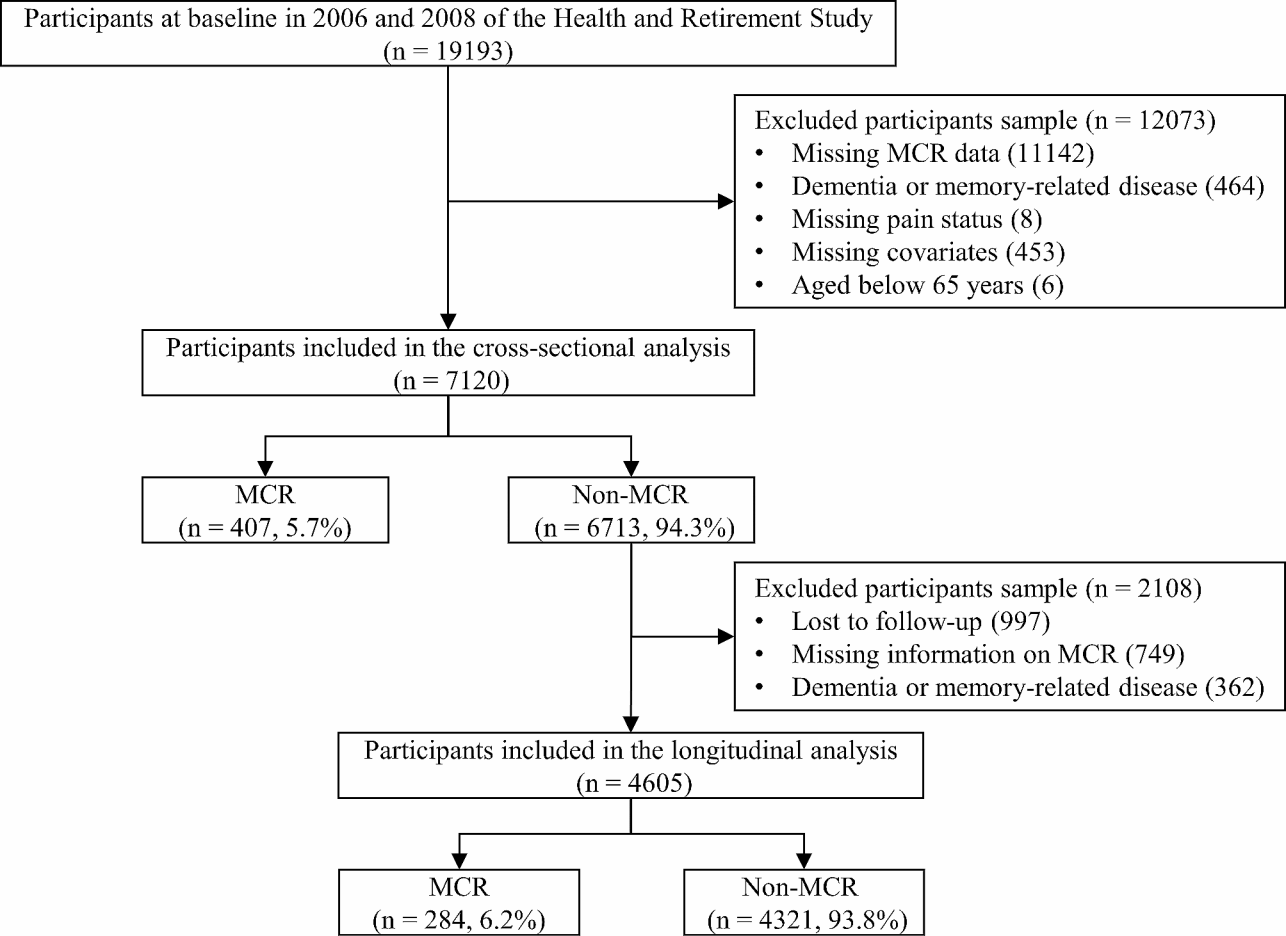
Flowchart of the screening process

The HRS is approved by the Institutional Review Board and conducted by the University of Michigan. It is a representative and national cohort study of people aged ≥ 50 years in the USA and sponsored by the National Institute on Aging (U01AG009740) [29]. Gait data were available for a random half-sample from both the 2006 and 2008 waves, separately, and were collected longitudinally every 4 years thereafter. In other words, the first random half-sample was drawn from the 2006 wave, and the second random half-sample was drawn from the 2008 wave. Therefore, we combined the data from 2006 and 2008 to establish the baseline. The first follow-up data were collected from the 2010 and 2012 waves, while the second follow-up data were collected from the 2014 and 2016 waves. The screening procedure is illustrated in Fig. 1. A total of 19,193 participants were included in the 2006 and 2008 waves. The following participants were excluded: missing MCR data (*n* = 11,142), with dementia or memory related diseases (*n* = 464), missing pain status (*n* = 8) and covariates (*n* = 453), and whose age less than 65 years (*n* = 6). Finally, 7120 participants were included at baseline. A total of 407 individuals with baseline MCR were excluded from the follow-up sample. In addition, those lost to follow-up (*n* = 997), without information on MCR (*n* = 749), and with dementia or memory-related diseases (*n* = 362) at follow-up were excluded. Finally, a total of 4605 participants were included in the follow-up.

### Pain interference

Some studies employ the Pain Interference subscale of the Brief Pain Inventory (BPI) to evaluate the degree of interfering pain on cognition [26, 28], while others ascertain the presence of interfering pain by inquiring whether pain influences individuals’ daily activities [22, 24, 30]. In this study, participants were first inquired if they were often troubled with pain. Those who responded affirmatively were then asked to rate their pain intensity (mild/moderate/severe) most of the time. Additionally, they were asked whether this pain interfered with their daily activities, such as household chores or work. According to these questions and a previous study, the states of pain interference were divided into 3 categories: interfering pain, non-interfering pain, and no pain [24].

### Motoric cognitive risk syndrome

The diagnostic criteria for MCR included subjective memory decline and slow gait, which were consistent with previous studies based on the HRS [11, 31–33]. In the HRS, subjective complaints in cognition were defined using the following two questions: (1) “How would you rate your memory at the present time? Would you say it is excellent, very good, good, fair, or poor?” (2) “Compared with the previous interview, would you say your memory is better now, about the same, or worse now than it was then?” Subjective complaints in cognition were determined if participants responded with “fair” or “poor” on the first question, or “worse” on the second question. Slow gait was defined as walking speed at least one standard deviation below sex- and age-specific averages. The cut-off values of slow gait in the HRS have been reported in recent research: < 75 years, male, 0.61 m/s; < 75 years, female, 0.54 m/s; ≥ 75 years, male, 0.48 m/s; ≥ 75 years, female, 0.42 m/s [11].

### Cognitive function

Cognitive function was assessed using a modified version of the Telephone Interview for Cognitive Status (TICS-m) in the HRS [34]. The TICS-m includes three types of cognitive tasks with a maximum score of 27: immediate and delayed memory, backward counting, and serial 7s. Based on the TICS-m scores, participants’ cognitive function can be categorized into three groups: normal (12 to 27 points), MCI (mild cognitive impairment) without dementia (7 to 11 points), and dementia (0 to 6 points) [34]. In addition, participants were asked whether they were diagnosed with dementia or memory-related diseases. Those who had dementia or memory-related diseases were excluded from our study.

### Covariates

Sociodemographic factors included sex, age, race and ethnicity, and educational level (defined as high-level if they had 12 years of education or more) [35]. The healthy conditions and behavioral variables consisted of obesity, smoking and drinking status, comorbidities, depression, and physical inactivity. Obesity was defined as having a body mass index (BMI) of ≥ 30 kg/m^2^. Tobacco and alcohol use were classified into three categories: never use, ever use, and current use. Comorbidities consisted of self-reported vascular diseases (heart disease, stroke, hypertension, and diabetes), arthritis, lung disease, and cancer. Physical inactivity was defined as participating in vigorous-intensity physical activities once a week or less [36]. Depression was defined as having a score ≥ 3 on the modified 8-item Centers for Epidemiologic Studies Depression Scale [37].

### Statistical analysis

Logistic and Cox regression were used to determine the associations between the states of pain interference and MCR at baseline and follow-up. Incident MCR event was defined as the first diagnosis of MCR during follow-up. Individuals without MCR were censored in their last evaluation. In all regression analyses, Model 1 was adjusted for sociodemographic factors, while Model 2 was additionally adjusted for healthy conditions and behaviors as well as cognitive function. Variables that violated the proportional hazards (PH) assumption, as determined by Schoenfeld residuals [38] were analyzed as time-dependent variables.

Three types of sensitivity analyses were conducted in this study. MCR and MCI are both intermediate conditions between natural aging and dementia [6, 7, 39]. Individuals with MCR may be combined with MCI [40]. To reduce the impact of MCI on self-reported measures, we excluded participants with MCI (TICS-m score of 7–11). Second, we excluded individuals with any components of MCR (subjective complaints in cognition or slow gait) from the non-MCR group. Third, individuals with non-interfering pain at baseline but developed interfering pain at follow-up were excluded from the longitudinal analysis. All analyses were performed on IBM SPSS 26.0 and R 4.3.1. This study was conducted in accordance with the STROBE guidelines (Additional file 1).

## Results

Table 1 shows the characteristics of participants according to the states of pain interference at baseline, which included 7120 older adults (average age of 74.6 years, 56.8% females). The prevalences of non-interfering and interfering pain were 13.1% and 18.0%, respectively. Significant differences were observed in comorbidities, obesity, education level, drinking status, physical activity, depression, and cognitive function across the three groups (Table 1). The excluded participants were older, had lower educational levels, and had poorer overall health (depression, the number of comorbidities, physical activities, and cognitive function) in both cross-sectional and longitudinal analyses (Table S1 and Table S2).

**Table 1:** Baseline participant characteristics according to pain status (*n* = 7120)

Characteristic	Overall	No Pain	Non-interfering Pain	Interfering Pain	*p*
	*n* = 7120	*n* = 4903 (68.9%)	*n* = 935 (13.1%)	*n* = 1282 (18.0%)	
**Age, years, mean (SD)**	74.6 (6.7)	74.7 (6.7)	74.3 (6.8)	74.5 (6.7)	0.210
**Female, n(%)**	4,041 (56.8%)	2,680 (54.7%)	515 (55.1%)	846 (66.0%)	**< 0.001**
**Race/ethnicity, n(%)**					0.052
Non-Hispanic White	5,783 (81.2%)	3,953 (80.6%)	776 (83.0%)	1,054 (82.2%)	
Non-Hispanic Black	738 (10.4%)	546 (11.1%)	85 (9.1%)	107 (8.3%)	
Hispanic	487 (6.8%)	328 (6.7%)	62 (6.6%)	97 (7.6%)	
Other	112 (1.6%)	76 (1.6%)	12 (1.3%)	24 (1.9%)	
**High-level education, n(%)**	5,390 (75.7%)	3,789 (77.3%)	705 (75.4%)	896 (69.9%)	**< 0.001**
**Obesity, n(%)**	1,964 (27.6%)	1,210 (24.7%)	269 (28.8%)	485 (37.8%)	**< 0.001**
**Smoking status, n(%)**					0.363
Never smoke	3,064 (43.0%)	2,128 (43.4%)	389 (41.6%)	547 (42.7%)	
Former smoke	3,400 (47.8%)	2,334 (47.6%)	465 (49.7%)	601 (46.9%)	
Current smoke	656 (9.2%)	441 (9.0%)	81 (8.7%)	134 (10.5%)	
**Drinking status, n(%)**					**< 0.001**
Never drink	3,544 (49.8%)	2,346 (47.8%)	453 (48.4%)	745 (58.1%)	
Former drink	1,250 (17.6%)	843 (17.2%)	169 (18.1%)	238 (18.6%)	
Current drink	2,326 (32.7%)	1,714 (35.0%)	313 (33.5%)	299 (23.3%)	
**Comorbidities, mean (SD)** ^**a**^	2.2 (1.3)	2.0 (1.2)	2.4 (1.2)	2.8 (1.2)	**< 0.001**
**Depression, mean (SD)**	1,209 (17.0%)	572 (11.7%)	172 (18.4%)	465 (36.3%)	**< 0.001**
**Cognitive function, mean (SD)**	15.0 (3.8)	15.1 (3.8)	15.0 (3.8)	14.7 (3.7)	**0.009**
**Physical inactivity, n(%)**	5,439 (76.4%)	3,628 (74.0%)	702 (75.1%)	1,109 (86.5%)	**< 0.001**
**MCR at baseline, n(%)**	407 (5.7%)	223 (4.5%)	56 (6.0%)	128 (10.0%)	**< 0.001**

Abbreviations: SD, standard deviation; MCR, motoric cognitive risk syndrome

^a^The number of comorbidities: stroke, hypertension, diabetes, heart disease, cancer, lung disease, and arthritis

There were 407 (5.7%) participants with MCR at baseline (Table 1). The prevalence of MCR was higher among older adults with non-interfering pain (6.0%) and interfering pain (10.0%) compared to those who had no pain (4.5%). The cross-sectional associations between different states of pain interference and MCR can be seen in Table 2. Interfering pain was associated with a greater likelihood of MCR, even after adjusting for sociodemographic factors (Model 1; OR = 2.36, 95% CI = 1.86–2.98; *p* < 0.001) and further adjusting for healthy conditions and behaviors, and cognitive function (Model 2; OR = 1.51, 95% CI = 1.17–1.95; *p* = 0.001).

**Table 2:** Cross-sectional association between pain status and MCR

Pain status	Event N	Model 1^a^		Model 2^b^	
		OR (95%CI)	*p*	OR (95%CI)	*p*
No Pain	223	—		—	
Non-interfering Pain	56	1.38 (1.01, 1.87)	**0.038**	1.18 (0.86, 1.60)	0.301
Interfering Pain	128	2.36 (1.86, 2.98)	**< 0.001**	1.51 (1.17, 1.95)	**0.001**

Abbreviations: MCR, motoric cognitive risk syndrome; OR, odds ratio; CI, confidence interval

^a^Model 1: Adjusted for demographic variables (age, sex, race/ethnicity, education);

^b^Model 2: Model 1 plus cognitive function (TICS-m), healthy conditions and behaviors (obesity, smoking status, drinking status, depression, physical activity and number of comorbidities)

Except for a single sensitivity analysis where the baseline cognitive function did not meet the PH assumption, all other analyses complied. The Cox regression analysis included 4605 older adults without MCR at baseline. During 8 years of follow-up, 284 (6.2%) participants developed incident MCR (Fig. 1). The risk of MCR significantly increased in individuals with non-interfering pain and those with interfering pain after adjusting for all covariates (Table 3; non-interfering pain: HR = 1.48, 95% CI = 1.06–2.08, *p* = 0.021; interfering pain: HR = 2.02, 95% CI = 1.52–2.69; *p* < 0.001).

**Table 3:** Longitudinal analysis of pain status and MCR

Pain status	Event N	Model 1^a^		Model 2^b^	
		HR (95%CI)	*p*	HR (95%CI)	*p*
No Pain	154	—		—	
Non-interfering Pain	45	1.65 (1.19, 2.31)	**0.003**	1.48 (1.06, 2.08)	**0.021**
Interfering Pain	85	2.79 (2.14, 3.65)	**< 0.001**	2.02 (1.52, 2.69)	**< 0.001**

Abbreviations: MCR, motoric cognitive risk syndrome; HR, hazard ratio; CI, confidence interval

^a^Model 1: Adjusted for demographic variables (age, sex, race/ethnicity, education)

^b^Model 2: Model 1 plus cognitive function (TICS-m), healthy conditions and behaviors (obesity, smoking status, drinking status, depression, physical activity and number of comorbidities)

**Fig. 2:**
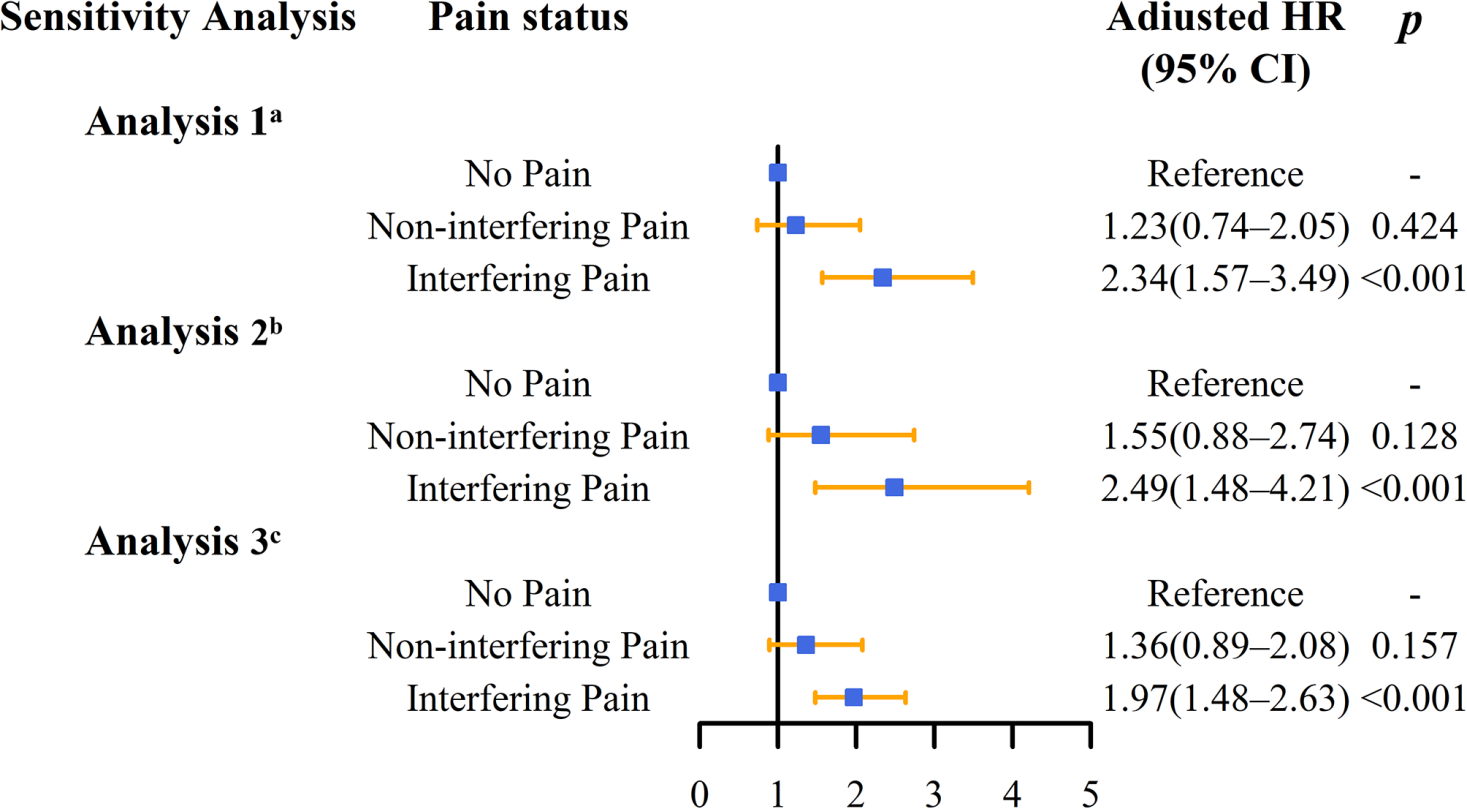
Summary of longitudinal sensitivity analyses. ^a^Excluding participants with MCI without dementia; ^b^Excluding participants with any of the MCR components (subjective complaints in cognition complaints or slow gait); ^c^Excluding participants with non-interfering pain at baseline but with interfering pain at follow-up. HR, hazard ratio; CI, confidence interval

Three types of sensitivity analyses were conducted. After excluding participants with MCI, the association between interfering pain and MCR at baseline (Table S3; OR = 1.62, 95% CI = 1.17–2.22; *p* = 0.003) and incident MCR (Fig. 2, Table S4; HR = 2.34, 95% CI = 1.57–3.49; *p* < 0.001) remained significant. Likewise, after excluding individuals with components of MCR from the non-MCR group, the association between interfering pain and concurrent MCR (Table S5, OR = 2.00, 95% CI = 1.50–2.64; *p* < 0.001) and incident MCR (Fig. 2, Table S6, HR = 2.49, 95% CI = 1.48–4.21; *p* < 0.001) persisted. Lastly, the correlation between interfering pain and the risk of incident MCR (Fig. 2, Table S7, HR = 1.97, 95% CI = 1.48–2.63; *p* < 0.001) remained robust even after excluding individuals with non-interfering pain at baseline but developed interfering pain at follow-up. However, non-interfering pain was not significantly related to MCR in either cross-sectional or longitudinal sensitivity analyses (Table S3, Table S5, Fig. 2).

## Discussion

In this population-based study of older people aged ≥ 65 years, we examined the correlation of different states of pain interference with concurrent and incident MCR. Nearly 30% of older adults were often troubled by pain at baseline. As hypothesized, the states of pain interference were linked to MCR at baseline and 8-year follow-up. The sensitivity analyses reinforced this finding. This study provides a new perspective on the association between pain and MCR.

During the 8-year follow-up, interfering pain approximately doubled the risk of developing MCR and was more strongly correlated with an increased risk of MCR versus non-interfering pain. Interfering pain was still significantly associated with a higher risk of MCR in all sensitivity analyses, which underscored the robustness of the association. Non-interfering pain was significantly associated with MCR in our longitudinal analysis, which is inconsistent with the results of the cross-sectional and additional analyses. Pain is a dynamic and subjective experience [21, 41]. Therefore, the states of pain interference would change during the follow-up period. We conducted an additional analysis that excluded individuals with non-interfering pain at baseline but developed interfering pain at follow-up, which revealed that non-interfering pain did not increase the risk of MCR. Thus, non-interfering pain might not pose a risk for MCR.

To the best of our knowledge, although no studies have examined the link between interfering pain and MCR, previous studies have associated interfering pain with cognitive decline. In a study of Puerto Rican adults aged ≥ 60 years, increased interfering pain at follow-up was associated with incident cognitive decline [24]. Another study of Mexican-Americans aged ≥ 80 years also reported that interfering pain was related to cognitive impairment [30]. It has been reported that either subjective memory decline or slow gait, both key characteristics of MCR, may precede MCI by more than a decade [42, 43]. Investigating the correlation between interfering pain and MCR rather than MCI may facilitate the prevention of cognitive decline at an earlier stage. Furthermore, given the simpler assessment of MCR, it might be more beneficial to utilize MCR as a cognitive screening tool in pain management. However, individuals with MCR may also have MCI [40]. Consequently, we excluded individuals with MCI, and the results remained robust.

An important reason why interfering pain is associated with an increased risk of MCR might be that it demands more attentional resources than non-interfering pain and no pain. According to the interruptive pain model, pain competes for individuals’ limited attentional resources, thereby interfering with basic tasks that demand attention [44]. Interfering pain has also been reported to be associated with poorer complex attention in older adults. Impaired attention may lead to a decline in memory and executive function [28]. Meanwhile, a decline in executive function is reported to be associated with slow gait [45]. Consequently, we speculate that interfering pain may increase the risk of MCR by affecting attention and executive function. Furthermore, overlapping brain changes may also be an important reason for the correlation between interfering pain and MCR. Pain can lead to structural and functional changes in the prefrontal cortex [46, 47], which is involved in emotional and cognitive processing [46], executive functions [48], and pain processing [48, 49]. Recent studies have also reported that individuals with MCR have a smaller prefrontal cortex volume [8, 9]. In summary, the interruptive pain model and brain changes may explain the relationship between interfering pain and MCR.

Our study has several strengths and important clinical implications. First, the combination of cross-sectional and longitudinal analyses enhances the reliability of the results. Second, this study concentrates on different states of pain interference, which serves as an important supplement to help understand the relationship between pain and MCR more comprehensively. Our results emphasize the significance of managing interfering pain in maintaining cognitive function. We found that interfering pain may increase the risk of MCR, which is a pre-dementia status and related to a series of adverse outcomes. Reporting interfering pain may be an important indicator of early cognitive changes in older adults. Treatment for interfering pain may help improve cognitive performance because the impact of pain, including that on the brain, is reversible after adequate treatment [48, 50, 51]. Therefore, interfering pain may be a therapeutic target for MCR. Early identification and timely treatment of interfering pain may reduce the risk of MCR and associated adverse outcomes such as dementia, physical impairment, and mortality. Additionally, the assessment of MCR does not require trained personnel or specialized equipment [20]. It facilitates easier and more regular monitoring of cognitive function in clinical- and home-based pain management. Ultimately, it helps to evaluate the effect of pain treatment and promptly adjust the treatment plan.

This study has several limitations. First, there could have been selection bias by excluding individuals who were lost to follow-up and without information on pain interference and MCR. The overall health status of the excluded individuals was worse; thus, it remains uncertain whether our findings can be generalized to populations with poorer health conditions. Second, information regarding the use of pain medications was unknown. Pain medications such as opioids may affect both pain perception and cognitive performance [52]. Third, this study was limited to investigating the association between the interference of pain with daily activities and MCR. It is recommended that future research broadens the scope of assessment by employing more comprehensive instruments. For instance, the Pain Interference subscale of the BPI, provides information about how much pain interferes with the affective and activity subdimensions [53]. Finally, it is difficult to establishing causal associations between interfering pain and MCR based only on observational data; additional experimental studies are needed to verify our results. Specifically, future studies should focus on effective pain management for individuals experiencing concurrent interfering pain and MCR. The effect of the alleviation of interfering pain on the cognitive and physical functions of individuals with MCR could then be observed.

## Conclusions

Individuals with interfering pain have a higher risk of developing MCR than those with non-interfering pain or without pain. Interfering pain may be a modifiable risk factor for developing MCR. To further investigate the link between pain interference and MCR, future research should subdivide pain interference into more specific items, consider primary and secondary pain, the duration and location of pain, and examine the use of pain medications. Additionally, as slow gait is one of the key characteristics of MCR, exploring the brain function of individuals with interfering pain during real-time walking using neuroimaging techniques such as functional near-infrared spectroscopy may reveal the brain mechanisms associated with the correlation between interfering pain and MCR.

### Electronic supplementary material

Below is the link to the electronic supplementary material.

Supplementary Material 1

## Data Availability

The dataset can be download from the official website of the HRS (https://hrs.isr.umich.edu/).

## Abbreviations

MCR: Motoric cognitive risk syndrome
HRS: Health and Retirement Study
TICS-m: Modified version of the Telephone Interview for Cognitive Status
MCI: Mild cognitive impairment
BMI: Body mass index
