# Supplementary material for: Association between pain interference and motoric cognitive risk syndrome in older adults: a population-based cohort study
Source: BMC Geriatr. 2024 May 17;24:437. doi: 10.1186/s12877-024-04974-7 (PMC11102256; doi:10.1186/s12877-024-04974-7)
Supplement: Supplementary file 1 — Supplementary Material 1 [file 12877_2024_4974_MOESM1_ESM.docx]

**SUPPLEMENT MATERIAL**

[STROBE Statement—Checklist of items that should be included in reports of *cohort studies* 1](#_Toc158131025)

[Supplementary Table S1 Baseline characteristics of participants excluded vs included from baseline sample 3](#_Toc158131026)

[Supplementary Table S2 Baseline characteristics of participants excluded vs included from follow-up sample 4](#_Toc158131027)

[Supplementary Table S3 Associations between pain status and MCR after excluding participants with MCI at baseline 5](#_Toc158131028)

[Supplementary Table S4 Associations between pain status and MCR after excluding participants with MCI at follow-up 6](#_Toc158131029)

[Supplementary Table S5 Associations between pain status and MCR after excluding participants with any of the MCR components from non-MCR individuals at baseline 7](#_Toc158131030)

[Supplementary Table S6 Associations between pain status and MCR after excluding participants with any of the MCR components from non-MCR individuals at follow-up 8](#_Toc158131031)

[Supplementary Table S7 Associations between pain status and MCR after excluding participants with non-interfering pain at baseline but developed interfering pain at follow-up 9](#_Toc158131032)

# STROBE Statement—Checklist of items that should be included in reports of *cohort studies*

|  | **Item No** | **Recommendation** | | **Page No** |
| --- | --- | --- | --- | --- |
| **Title and abstract** | 1 | (*a*) Indicate the study’s design with a commonly used term in the title or the abstract | | 1 |
|  |  | (*b*) Provide in the abstract an informative and balanced summary of what was done and what was found |  | 1 |
| **Introduction** | | |  |  |
| Background/rationale | 2 | Explain the scientific background and rationale for the investigation being reported |  | 2 |
| Objectives | 3 | State specific objectives, including any prespecified hypotheses |  | 2 |
| **Methods** | | |  |  |
| Study design | 4 | Present key elements of study design early in the paper |  | 2 |
| Setting | 5 | Describe the setting, locations, and relevant dates, including periods of recruitment, exposure, follow-up, and data collection |  | 2 |
| Participants | 6 | (*a*) Give the eligibility criteria, and the sources and methods of selection of participants. Describe methods of follow-up |  | 2  Figure 1 |
|  |  | (*b*) For matched studies, give matching criteria and number of exposed and unexposed |  |  |
| Variables | 7 | Clearly define all outcomes, exposures, predictors, potential confounders, and effect modifiers. Give diagnostic criteria, if applicable |  | 2, 3, 4 |
| Data sources/ measurement | 8* | For each variable of interest, give sources of data and details of methods of assessment (measurement). Describe comparability of assessment methods if there is more than one group |  | 2, 3, 4 |
| Bias | 9 | Describe any efforts to address potential sources of bias |  | 4 |
| Study size | 10 | Explain how the study size was arrived at |  | Not applicable |
| Quantitative variables | 11 | Explain how quantitative variables were handled in the analyses. If applicable, describe which groupings were chosen and why |  | 2, 3, 4 |
| Statistical methods | 12 | (*a*) Describe all statistical methods, including those used to control for confounding |  | 4 |
|  |  | (*b*) Describe any methods used to examine subgroups and interactions |  | Not applicable |
|  |  | (*c*) Explain how missing data were addressed |  | Not applicable |
|  |  | (*d*) If applicable, explain how loss to follow-up was addressed |  | Not applicable |
|  |  | (*e*) Describe any sensitivity analyses |  | 4 |
| **Results** | | |  |  |
| Participants | 13* | (a) Report numbers of individuals at each stage of study—eg numbers potentially eligible, examined for eligibility, confirmed eligible, included in the study, completing follow-up, and analysed |  | 4, 5  Figure 1 |
|  |  | (b) Give reasons for non-participation at each stage |  | Not applicable |
|  |  | (c) Consider use of a flow diagram |  | Figure 1 |
| Descriptive data | 14* | (a) Give characteristics of study participants (eg demographic, clinical, social) and information on exposures and potential confounders |  | Table 1 |
|  |  | (b) Indicate number of participants with missing data for each variable of interest |  | Figure 1 |
|  |  | (c) Summarise follow-up time (eg, average and total amount) |  | 5 |
| Outcome data | 15* | Report numbers of outcome events or summary measures over time |  | Not applicable |
| Main results | 16 | (*a*) Give unadjusted estimates and, if applicable, confounder-adjusted estimates and their precision (eg, 95% confidence interval). Make clear which confounders were adjusted for and why they were included |  | 5 |
|  |  | (*b*) Report category boundaries when continuous variables were categorized |  | 3, 4 |
|  |  | (*c*) If relevant, consider translating estimates of relative risk into absolute risk for a meaningful time period |  | Not applicable |
| Other analyses | 17 | Report other analyses done—eg analyses of subgroups and interactions, and sensitivity analyses |  | 5 |
| **Discussion** | | |  |  |
| Key results | 18 | Summarise key results with reference to study objectives |  | 5, 6 |
| Limitations | 19 | Discuss limitations of the study, taking into account sources of potential bias or imprecision. Discuss both direction and magnitude of any potential bias |  | 7 |
| Interpretation | 20 | Give a cautious overall interpretation of results considering objectives, limitations, multiplicity of analyses, results from similar studies, and other relevant evidence |  | 6 |
| Generalisability | 21 | Discuss the generalisability (external validity) of the study results |  | 7 |
| **Other information** | | |  |  |
| Funding | 22 | Give the source of funding and the role of the funders for the present study and, if applicable, for the original study on which the present article is based |  | 7 |

*Give information separately for exposed and unexposed groups.

**Note:** An Explanation and Elaboration article discusses each checklist item and gives methodological background and published examples of transparent reporting. The STROBE checklist is best used in conjunction with this article (freely available on the Web sites of PLoS Medicine at http://www.plosmedicine.org/, Annals of Internal Medicine at http://www.annals.org/, and Epidemiology at http://www.epidem.com/). Information on the STROBE Initiative is available at http://www.strobe-statement.org.

| **Characteristic** | **Overall, n = 13082** | **Excluded, n = 5962(45.6%)** | **Included, n = 7120(54.4%)** | ***p*** |
| --- | --- | --- | --- | --- |
| **Age, years, mean (SD)** | 75.4 (7.9) | 76.4 (9.0) | 74.6 (6.7) | **<0.001** |
| **Female, n(%)** | 7,543 (57.7%) | 3,502 (58.7%) | 4,041 (56.8%) | **0.022** |
| **Race/ethnicity, n(%)** |  |  |  | **<0.001** |
| Non-Hispanic White | 9,994 (76.4%) | 4,211 (70.7%) | 5,783 (81.2%) |  |
| Non-Hispanic Black | 1,780 (13.6%) | 1,042 (17.5%) | 738 (10.4%) |  |
| Hispanic | 1,064 (8.1%) | 577 (9.7%) | 487 (6.8%) |  |
| Other | 242 (1.9%) | 130 (2.2%) | 112 (1.6%) |  |
| **High-level education, n(%)** | 9,022 (69.0%) | 3,632 (60.9%) | 5,390 (75.7%) | **<0.001** |
| **Obesity, n(%)** | 3,539 (27.5%) | 1,575 (27.3%) | 1,964 (27.6%) | 0.734 |
| **Smoking status, n(%)** |  |  |  | **0.032** |
| Never smoke | 5,513 (42.5%) | 2,449 (41.9%) | 3,064 (43.0%) |  |
| Former smoke | 6,177 (47.7%) | 2,777 (47.5%) | 3,400 (47.8%) |  |
| Current smoke | 1,273 (9.8%) | 617 (10.6%) | 656 (9.2%) |  |
| **Drinking status, n(%)** |  |  |  | **<0.001** |
| Never drink | 7,365 (56.4%) | 3,821 (64.3%) | 3,544 (49.8%) |  |
| Former drink | 2,015 (15.4%) | 765 (12.9%) | 1,250 (17.6%) |  |
| Current drink | 3,684 (28.2%) | 1,358 (22.8%) | 2,326 (32.7%) |  |
| **Comorbidities, mean (SD)^a^** | 2.3 (1.3) | 2.4 (1.4) | 2.2 (1.3) | **<0.001** |
| **Depression, mean (SD)** | 2,544 (21.6%) | 1,335 (28.6%) | 1,209 (17.0%) | **<0.001** |
| **Cognitive function, mean (SD)** | 14.5 (4.2) | 9.6 (5.4) | 15.0 (3.8) | **<0.001** |
| **Physical inactivity, n(%)** | 10,564 (80.8%) | 5,125 (86.2%) | 5,439 (76.4%) | **<0.001** |

# Supplementary Table S1 Baseline characteristics of participants excluded vs included from baseline sample

Abbreviations: SD, standard deviation

^a^The number of comorbidities: stroke, hypertension, diabetes, heart disease, cancer, lung disease, and arthritis.

# Supplementary Table S2 Baseline characteristics of participants excluded vs included from follow-up sample

| **Characteristic** | **Overall, n= 6713** | **Excluded, n = 2108(31.4%)** | **Included, n = 4605(68.6%)** | ***p*** |
| --- | --- | --- | --- | --- |
| **Age, years, mean (SD)** | 74.5 (6.7) | 77.2 (7.4) | 73.3 (5.9) | **<0.001** |
| **Female, n(%)** | 3,803 (56.7%) | 1,143 (54.2%) | 2,660 (57.8%) | **0.007** |
| **Race/ethnicity, n(%)** |  |  |  | **<0.001** |
| Non-Hispanic White | 5,528 (82.3%) | 1,655 (78.5%) | 3,873 (84.1%) |  |
| Non-Hispanic Black | 655 (9.8%) | 258 (12.2%) | 397 (8.6%) |  |
| Hispanic | 423 (6.3%) | 156 (7.4%) | 267 (5.8%) |  |
| Other | 107 (1.6%) | 39 (1.9%) | 68 (1.5%) |  |
| **High-level education, n(%)** | 5,174 (77.1%) | 1,442 (68.4%) | 3,732 (81.0%) | **<0.001** |
| **Obesity, n(%)** | 1,814 (27.0%) | 516 (24.5%) | 1,298 (28.2%) | **0.001** |
| **Smoking status, n(%)** |  |  |  | **<0.001** |
| Never smoke | 2,906 (43.3%) | 829 (39.3%) | 2,077 (45.1%) |  |
| Former smoke | 3,185 (47.4%) | 1,037 (49.2%) | 2,148 (46.6%) |  |
| Current smoke | 622 (9.3%) | 242 (11.5%) | 380 (8.3%) |  |
| **Drinking status, n(%)** |  |  |  | **<0.001** |
| Never drink | 3,274 (48.8%) | 1,178 (55.9%) | 2,096 (45.5%) |  |
| Former drink | 1,196 (17.8%) | 311 (14.8%) | 885 (19.2%) |  |
| Current drink | 2,243 (33.4%) | 619 (29.4%) | 1,624 (35.3%) |  |
| **Comorbidities, mean (SD)^a^** | 2.2 (1.2) | 2.4 (1.3) | 2.1 (1.2) | **<0.001** |
| **Depression, mean (SD)** | 1,048 (15.6%) | 439 (20.8%) | 609 (13.2%) | **<0.001** |
| **Cognitive function, mean (SD)** | 15.1 (3.7) | 13.5 (3.7) | 15.9 (3.5) | **<0.001** |
| **Physical inactivity, n(%)** | 5,083 (75.7%) | 1,740 (82.5%) | 3,343 (72.6%) | **<0.001** |

Abbreviations: SD, standard deviation; MCR, motoric cognitive risk syndrome.

^a^The number of comorbidities: stroke, hypertension, diabetes, heart disease, cancer, lung disease, and arthritis.

# Supplementary Table S3 Associations between pain status and MCR after excluding participants with MCI at baseline

| **Pain status** | **Model 1**^a^ | | **Model 2**^b^ | |
| --- | --- | --- | --- | --- |
|  | **OR (95%CI)** | ***p*** | **OR (95%CI)** | ***p*** |
| No Pain | **—** |  | **—** |  |
| Non-interfering Pain | 1.57 (1.05, 2.27) | **0.021** | 1.32 (0.89, 1.94) | 0.158 |
| Interfering Pain | 2.63 (1.96, 3.52) | **<0.001** | 1.62 (1.17, 2.22) | **0.003** |

Abbreviations: MCR, motoric cognitive risk syndrome; MCI, mild cognitive impairment; OR, odds ratio; CI, confidence interval.

^a^Model 1: Adjusted for demographic variables (age, sex, race/ethnicity, education).

^b^Model 2: Model 1 plus cognitive function (TICS-m), healthy conditions and behaviors (obesity, smoking status, drinking status, depression, physical activity and number of comorbidities).

# Supplementary Table S4 Associations between pain status and MCR after excluding participants with MCI at follow-up

| **Pain status** | **Model 1**^a^ | | **Model 2**^b^ | |
| --- | --- | --- | --- | --- |
|  | **HR (95%CI)** | ***p*** | **HR (95%CI)** | ***p*** |
| No Pain | — |  | — |  |
| Non-interfering Pain | 1.41 (0.85, 2.34) | 0.183 | 1.23 (0.74, 2.05) | 0.424 |
| Interfering Pain | 3.41 (2.35, 4.96) | **<0.001** | 2.34 (1.57, 3.49) | **<0.001** |

Abbreviations: MCR, motoric cognitive risk syndrome; MCI, mild cognitive impairment; HR, hazard ratio; CI, confidence interval.

^a^Model 1: Adjusted for demographic variables (age, sex, race/ethnicity, education).

^b^Model 2: Model 1 plus cognitive function (TICS-m), healthy conditions and behaviors (obesity, smoking status, drinking status, depression, physical activity and number of comorbidities).

# Supplementary Table S5 Associations between pain status and MCR after excluding participants with any of the MCR components from non-MCR individuals at baseline

| **Pain status** | **Model 1**^a^ | | **Model 2**^b^ | |
| --- | --- | --- | --- | --- |
|  | **OR (95%CI)** | ***p*** | **OR (95%CI)** | ***p*** |
| No Pain | — |  | — |  |
| Non-interfering Pain | 1.49 (1.07, 2.05) | **0.014** | 1.30 (0.93, 1.81) | 0.121 |
| Interfering Pain | 3.53 (2.74, 4.54) | **<0.001** | 2.00 (1.50, 2.64) | **<0.001** |

Abbreviations: MCR, motoric cognitive risk syndrome; OR, odds ratio; CI, confidence interval.

^a^Model 1: Adjusted for demographic variables (age, sex, race/ethnicity, education).

^b^Model 2: Model 1 plus cognitive function (TICS-m), healthy conditions and behaviors (obesity, smoking status, drinking status, depression, physical activity and number of comorbidities).

# Supplementary Table S6 Associations between pain status and MCR after excluding participants with any of the MCR components from non-MCR individuals at follow-up

| **Pain status** | **Model 1**^a^ | | **Model 2**^b^ | |
| --- | --- | --- | --- | --- |
|  | **HR (95%CI)** | ***p*** | **HR (95%CI)** | ***p*** |
| No Pain | — |  | — |  |
| Non-interfering Pain | 1.77 (1.01, 3.09) | **0.045** | 1.55 (0.88, 2.74) | 0.128 |
| Interfering Pain | 3.60 (2.20, 5.91) | **<0.001** | 2.49 (1.48, 4.21) | **<0.001** |

Abbreviations: MCR, motoric cognitive risk syndrome; HR, hazard ratio; CI, confidence interval.

^a^Model 1: Adjusted for demographic variables (age, sex, race/ethnicity, education).

^b^Model 2: Model 1 plus cognitive function (TICS-m), healthy conditions and behaviors (obesity, smoking status, drinking status, depression, physical activity and number of comorbidities).

# Supplementary Table S7 Associations between pain status and MCR after excluding participants with non-interfering pain at baseline but developed interfering pain at follow-up

| **Pain status** | **Model 1**^a^ | | **Model 2**^b^ | |
| --- | --- | --- | --- | --- |
|  | **HR (95%CI)** | ***p*** | **HR (95%CI)** | ***p*** |
| No Pain | — |  | — |  |
| Non-interfering Pain | 1.45 (0.95, 2.21) | 0.085 | 1.36 (0.89, 2.08) | 0.157 |
| Interfering Pain | 2.79 (2.13, 3.64) | **<0.001** | 1.97 (1.48, 2.63) | **<0.001** |

Abbreviations: MCR, motoric cognitive risk syndrome; HR, hazard ratio; CI, confidence interval.

^a^Model 1: Adjusted for demographic variables (age, sex, race/ethnicity, education).

^b^Model 2: Model 1 plus cognitive function (TICS-m), healthy conditions and behaviors (obesity, smoking status, drinking status, depression, physical activity and number of comorbidities).
